# Hyperkalemic Periodic Paralysis: Case Report with a SCNA4 Gene Mutation and Literature Review

**DOI:** 10.1155/2020/8843410

**Published:** 2020-10-16

**Authors:** Manuela Quiroga-Carrillo, Cristian Correa-Arrieta, Fernando Ortiz-Corredor, Fernando Suarez-Obando

**Affiliations:** ^1^Instituto Roosevelt, Bogotá, Colombia; ^2^Group of Neuromuscular Diseases, Instituto Roosevelt, Bogotá, Colombia; ^3^Universidad Nacional de Colombia, Bogotá, Colombia; ^4^Instituto de Genética Humana, Pontificia Universidad Javeriana, Bogotá, Colombia

## Abstract

Hyperkalemic periodic paralysis is a rare musculoskeletal disorder characterized by episodic muscle weakness associated with hyperkalemia. It is a channelopathy associated with point mutations in the SCNA4 gene, with an autosomal dominant pattern of inheritance. We report the case of a 39-year-old patient with a picture with onset at six years of age, consisting of episodes of weakness caused by physical activity and intercurrent infectious processes, in whom a point mutation was found in the SCNA4 gene, not previously reported in the literature.

## 1. Introduction

Hyperkalemic periodic paralysis (HYPP) (OMIM: 170500, ORPHANET: ORPHA: 682) is an autosomal dominant muscle channelopathy with nearly complete penetrance. It is characterized by recurrent episodes of muscle weakness associated with elevated levels of blood potassium [1, 2]. It was first described by Tyler et al. in 1951, in a study of 7 generations of individuals with typical periodic paralysis in the absence of hypokalemia [3]. The disease affects approximately 1 in every 200,000 individuals [4]. Although typically hereditary, multiple de novo mutations have been reported [2].

We report the case of a 39-year-old patient with documented hyperkalemic periodic paralysis, probably secondary to variant c. 4483A > G (p. Ile1495Val) in the *SCN4A* (17q23.3) gene, a variant not previously reported in the literature.

## 2. Case Report

The patient is a 39-year-old male with a clinical picture that began at six years of age. He presented muscular weakness of progressive establishment, with a frequency of one episode per month, which worsened with intense physical exertion, associated with intercurrent febrile symptoms that limited his ability to perform physical activity. The crisis was variable in intensity, some of them presenting only with mild to moderate weakness and some others with complete paralysis. During infancy, nonpotassium alteration was documented during the crisis. Although the situation was periodic, the patient did not suffer motor development delay, and he achieved all his milestones without difficulty.

Crisis worsens in adulthood, being more severe and related to physical exercise of moderate intensity. No other triggers were identified as drugs or anesthetic events. Many of the episodes required short-term hospitalizations lasting two or three days. During hospitalizations, several electrocardiograms, brain MRI, and toxic profiles were normal. Other paraclinical tests, such as renal and thyroid function, hepatic function, screening for Pompe disease, and levels of lactic acid and pyruvate, were within normal limits.

During the last crisis, the only positive findings were mild to moderately increased CPK levels (values ranged between 209 and 873 U/L). Also, potassium measurements reached levels until 5.3 mEq/L in blood samples analyzed during the weakness and paralysis episodes. The electromyography study performed by multiMUP (multimotor unit potential) showed no abnormalities. These preliminary findings were interpreted as compatible with muscle fiber disease.

He did not present with important personal antecedents, although he stated that a paternal great-uncle died at age 50 due to a condition associated with motor impairment; however, in that case, there was no specific diagnosis.

**Figure 1:**
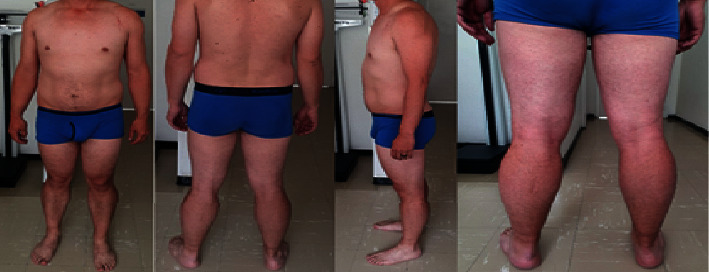
Clinically evident gastrocnemius hypertrophy. Normal muscular trophism in the rest of the body.

On physical examination, the patient presented weakness of proximal predominance in all the four extremities, with 4+/5 strength in shoulder abductors and adductors, elbow flexors, and extenders; in lower extremities, he presented 4/5 strength in hip flexors and extenders, as well as knee flexors. The rest of the muscle groups were within normal limits. He also showed four limb hyporeflexia, normal muscle tone, and gastrocnemius hypertrophy (see Figure 1).

A muscle biopsy performed at sixteen years old showed isolated muscle necrosis with reparative changes. A second biopsy performed in adulthood showed changes of denervation, regenerating fibers, increased lipid deposition, and intracytoplasmic glycogen deposits.

Considering the histopathological findings of the second biopsy, an NGS (Next-Generation Sequencing) genetic diagnosis panel was requested, which included the PYGM, PGAM1, PFKM, and LDHA genes for glycogen storage disease. The NGS panel showed no alterations.

Later, due to the course of the disease and the presence of relapses suggestive of periodic paralysis associated with abnormal potassium levels, it was decided to request a new genetic panel for periodic paralysis, which included the CACN1S, KCNA2, KCNJ2, KCNJ5, and SCN4A genes.

The panel demonstrated a missense variant in *SCN4A* c.4483A > G (p.Ile1495Val.). This variant has not been previously reported in the literature. However, it is considered likely pathogenic according to the ACMG/ACP standards [5] and Sherloc criteria [6]. None of the parents have the same variant, and none of them or any other family members were symptomatic.

Based on clinical presentation, the increased levels of potassium during the crisis, and the likely pathogenic variant found in gene *SCN4A*, we decided to start treating the patient as a case of hyperkalemic paralysis, prescribing hydrochlorothiazide 25 mg daily, subsequently increasing it 50 mg daily. A comprehensive rehabilitation plan tailored to the specific pathology was also established and specific diet recommendations. With this treatment, the patient presented a marked improvement in both weakness symptoms and functionality, and the frequency of weakness episodes was reduced.

## 3. Discussion

Hyperkalemic periodic paralysis (HYPP) is an autosomal dominant muscle channelopathy characterized by recurrent and transient episodes of flaccid paralysis associated with hyperkalemia. The symptoms of HYPP typically begin in the first decade of life. Patients have a crisis of mild to severe muscle weakness that lasts from minutes to several hours. The crisis is of morning predominance. Attacks are triggered or aggravated by the intake of potassium-rich foods, emotional stress, fasting, environmental cold, glucocorticoids, pregnancy, or rest after intense exercise [1]. The severity of the episodes varies significantly, and the same pathogenic variant could have variable expressivity within the affected families [2]. It often affects the thigh and calf muscles, which present hypertrophy. During attacks, reflexes may be diminished or absent, and, rarely, the respiratory and bulbar muscles may be affected. Sphincter muscles maintain tonicity during attacks. Sensitivity is not affected during the episodes, although some individuals may experience an aura of muscle paresthesia and discomfort before the onset of weakness [2, 7].

Hyperkalemic periodic paralysis has three clinically distinct manifestations: (1) without myotonia, (2) with clinical or electromyographic myotonia, or (3) with congenital paramyotonia. In all three forms, episodes of weakness occur in the same way [2, 6]. Electrical myotonia can be demonstrated on the electromyographic study in 50–75% of patients with HYPP, while less than 20% manifests clinically. In the latter, the myotonia is mild and can be provoked by percussion or voluntary contraction in the face, tongue, forearms, and thenar eminence.

During episodes, individuals may be in normokalemia or hyperkalemia. Potassium levels range from the upper limit of normality to values close to the cardiotoxic range. Usually, patients do not experience cardiac arrhythmias or respiratory failure due to hyperkalemia during weakness attacks. After these episodes, patients report muscle pain that lasts from hours to days in the involved muscle groups, with subsequent spontaneous recovery. In the period between weakness attacks, patients have normal potassium levels, sensation, reflexes, and strength without alterations, although over the years, permanent weakness could develop [2, 7].

From the genetic point of view, this pathology is caused by point mutations in the SCN4A gene. This gene encodes the alpha subunit of the skeletal muscle voltage-gated sodium channel Nav 1.4, which causes an alteration in the regular exchange of ions in the sarcolemma, consequently reducing the muscle contraction ability and producing weakness or paralysis [2]. Mutations in the SCN4A gene are associated with multiple neuromuscular disorders, including HYPP, normokalemic periodic paralysis, hypokalemic periodic paralysis, congenital paramiotony, potassium-aggravated myotonia, congenital myasthenic syndromes, and even heart rhythm pathologies such as Brugada syndrome; this denotes a wide allelic heterogeneity related to various neuromuscular phenotypes, which are part of the differential diagnosis [8]. Despite the phenotypic overlap of these pathologies, the clinical approach can differentiate them together with the paraclinical findings that indicate the electrolyte disorder during crises and the response to the medications used in the treatment [8, 9].

The differential diagnosis of HYPP includes conditions that share muscular symptoms such as paralysis and weakness and have a genetic basis; for instance, CACNA1A has been reported in association with periodic paralysis related to abnormal potassium levels [10, 11]. Mutations in the gene KCNA2 cause dominant-negative loss of function or a gain of function of the voltage-gated potassium channel, causing episodic ataxia associated with tremors, muscle twitching, and stiffening of the body [12]. KCNJ2 and KCNJ5 are related to cardio arrhythmic periodic paralysis (Andersen–Tawil syndrome) [13].

In the clinical case, a genetic variant (c.4483A > G p. Ile1495Val.) was found in the SCN4A gene, which presents in a patient with documented hyperkalemia during crises and who has had a favorable therapeutic response. Although this variant has not been previously reported in the literature, the coherence between the clinical picture and the identified genotype indicates the biological plausibility of the genotype-phenotype correlation for the identified variant. However, this finding must be confirmed with functional studies or with cases of other patients where both the variant and a clinical picture compatible with the bioinformatic prediction are documented.

Finally, regarding treatment, two approaches must be taken into account: (1) Acute treatment, which is performed with nonpharmacological measures, such as low-intensity exercise and carbohydrate intake, and also with the use of beta-2-agonist inhalers (such as salbutamol). (2) Preventive treatment is carried out with measures such as frequent carbohydrate intake, avoidance of fasting, intense exercise, and exposure to cold and potassium-rich foods. On the other hand, from the pharmacological point of view, one of the following drugs is used: dichlorphenamide (50 to 200 mg daily), acetazolamide (5–10 mg/kg/d in children and 125–1000 mg daily in adults), or hydrochlorothiazide (25–75 mg daily) [7, 11].

## 4. Conclusions

Recurrent episodes of muscle weakness must be a sign of suspecting paralysis associated with alterations in potassium levels. Electrolyte levels should be measured during a weakness crisis. The cause of paralysis is genetic in origin. In this case, a clinical picture of periodic paralysis associated with elevated potassium was documented. The patient has a likely pathogenic variant in the SCN4A gene. Mutations in this gene are associated with the clinical picture presented. The therapeutic response reinforces the idea of the relationship between the mutation as the cause of the clinical picture.

## Data Availability

The laboratory test and clinical data used to support the findings of this study are included within the article.
